# Effect of Mg/Si Ratio on the Bendability and Anisotropic Bend Behavior of Extruded 6000-Series Al Alloy

**DOI:** 10.3390/ma16093599

**Published:** 2023-05-08

**Authors:** Qi Zhang, Xiaochang Xu, Yu Wang

**Affiliations:** 1 School of Materials Science and Engineering, Central South University, Changsha 410083, China; 2 CYMA Precision Aluminum Co., Ltd., Ma’anshan 243000, China

**Keywords:** aluminum, bendability, texture, anisotropy, particles, fracture

## Abstract

Extruded Al-Mg-Si profiles applied in the automotive industry are required to achieve an appropriate combination of strength and bendability. In order to investigate the effect of Mg/Si ratio on the bendability and anisotropic bending behavior, AA6005 and AA6061C were extruded to 2 mm thick plates. More Goss texture and anisotropic particle clusters exist in AA6005 alloys with a low Mg/Si ratio, which leads to a high tendency of surface roughing and cracking and to strong anisotropy in their bendability. However, more low-angle grain boundaries, cubic texture and comparatively random distribution of particles exists in AA6061C alloys with a high Mg/Si ratio, which blunts the surface roughing and crack process. The surface undulation is the outcome of the strain-intense localization of several layers of grains in the vicinity of the outer elongated surface. The strain localization and surface undulation lead to shear band initiation near the valleys. Several cooperating micro-mechanisms in AA6005, including grain clusters with Goss and Cubic orientation, heterogeneously nucleated particles and grain boundary spatial arrangements, lead to the grain boundary decohesion along a shear direction. AA6005 shows for predominately intergranular fractures in nature, with some areas exhibiting grain boundary decohesion during bending in the TD. However, AA6061C shows a predominately transgranular in nature, with some areas exhibiting intergranular fracture, which is affected by shear band development.

## 1. Introduction

Al-Mg-Si alloy is a typical age-hardened aluminum alloy and can be hot-extruded to form complicated muti-chamber sections. Hence, Al-Mg-Si-extruded profiles with excellent crash performance are widely used for structural components such as bumpers, the front rail, pillars and side impact beams [1,2,3]. In contrast to the tensile test, the bend test is more suitable to evaluate the plastic of automotive structures during crashes, e.g., impacts or collisions [3,4,5]. However, extruded Al-Mg-Si alloys have strong anisotropy when bending and lower fracture resistance as strength increases [6,7]. Hence, there is a tendency to enhance the bendability of Al-Mg-Si alloys to further promote their applicability to various structural components within vehicles.

Recent publications have shown that the bendability of Al-Mg-Si alloys is limited by surface cracking, which is closely linked to the development of surface roughness and undulation on the outer elongated surface. Surface roughing during bending can be grouped into two forms of grain-scale: orange peel and ridging. Orange peel is characterized by out-of-plane displacement fields on the surface, which roughly maps the grain shape of the material. Ridges are present in the form of banded surface undulations along the bending axis [8,9]. Early studies indicate that the surface roughness is attributed to the local incompatibility of adjacent grains during plastic straining and depends on the grain size and crystallographic orientation [10,11]. More recent experimental studies focus on the influence of the spatial texture distribution on the surface ridging. It shows that soft (i.e., Cube, {111}[uvw]) and hard (i.e., Goss) grain clusters stimulate the strong heterogeneity of the surface strain and promote ridging behavior [12,13]. Shear band generally initiates at the points of strain localization and coincides with regions of high surface roughness. The constituent particles and micro-textures promote strain localization and shear band formation [14,15,16,17,18]. Some authors deem that the formation of shear band further facilitates the surface roughing and decreases the bendability [19,20,21].

The microstructural aspects that influence the strain localization, surface roughness and shear band development can be summed up as follows: (i) grain size and orientation; (ii) metallurgraphic texture; (iii) grain boundary precipitates and PFZ [22,23]; (iv) metallurgical aspects, i.e., primary particles heterogeneous nucleation. Hidetoshi [24] reported that bendability is influenced by second-phase particles and the formation of the share band. Zhenguo Li et al. [14] investigated the influence of the Mg and Si content on microstructure, crystallographic texture and bendability. Crack initiation was observed at the α-Al(FeMn)Si constituent particles, and the propagation of cracks proceeded by a combination of shear bands and void growth/coalescence. Generally, the fracture behavior when bending is an outcome of cooperation and competition among several micro-mechanisms [25]. In the early stage, Snilsberg et al. [6] revealed that the recrystallized alloys (e.g., 6060 and 7030) and the fibrous alloys (e.g., 6082 and 7003) both exhibit a distinctively lower value in the bending angle when the bending axis is parallel to the extrusion direction (ED) compared to the transverse direction (TD). It is speculated that the anisotropy in the bending angle is linked with the particle distribution, but no signs of further investigation are noticeable. Westermann et al. also found that the alignment of the primary particles give rise to the observed anisotropy, and the shear band formation is the major failure initiation mechanism during bending [26]. Recently, Shogo et al. [7] investigated the effect of local texture and residual stress on bendability, and their results indicate that the large local changes in the direction of the principal stress led to the anisotropic bending behavior.

The bendability and anisotropic bending behavior of extruded Al-Mg-Si alloys are still not well understood. To further clarify the bending anisotropy and enhance the bendability of extruded Al-Mg-Si alloys, a comprehensive experimental study was performed in the present work. Two alloys were extruded to obtain the comparative microstructure: (i) AA6005 alloy with excessive Si, and (ii) AA6061C alloy with excessive Mg. The purpose was to investigate the relationship between microstructure, surface roughing, strain localization, shear band development, crack initiation and fracture.

## 2. Materials and Methods

### 2.1. Material and Processing

**Figure 1:**
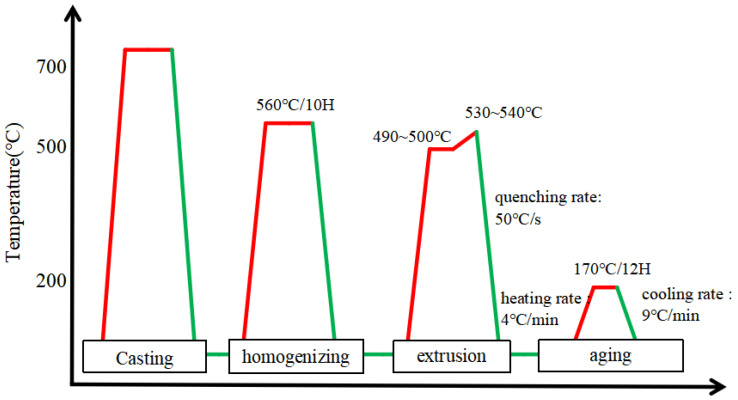
Schematic illustration of the thermal cycle during the processing.

The chemical compositions of AA6005 and AA6061C alloys are shown in Table 1. The two alloys have the same content of Mg + Si (1.35 wt%) to obtain the close value of yield (260–270 MPa). The entire thermal cycle that specimens experienced during processing is exhibited in Figure 1. The industrial-scale billet with 127 mm diameter were DC-cast. In preparation for the following hot extrusion, the cast billets were homogenized at 560 °C for a holding time of 12 h, and afterwards they were preheated to 500 °C [27]. Plates with a 2 mm thickness and 110 mm width were produced at a solutionizing temperature of 530 °C using a 1000-ton direct press. The plates were then quenched through the water bath to keep the alloying element in solid solution. Finally, the plates were aged at 170 °C for 12 h to achieve the T6 temper.

### 2.2. Mechanical Behavior Characterization

**Figure 2:**
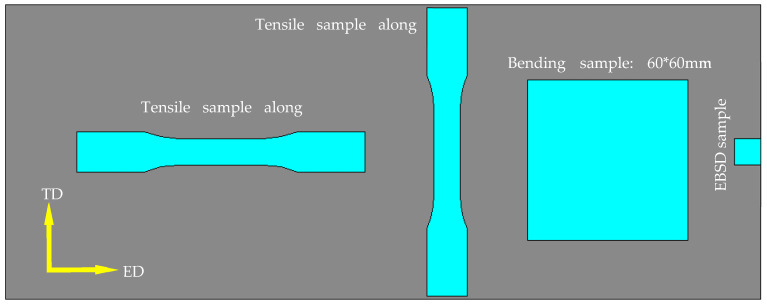
Schematic representation of specimens and the sampling solution from the extruded plate.

**Figure 3:**
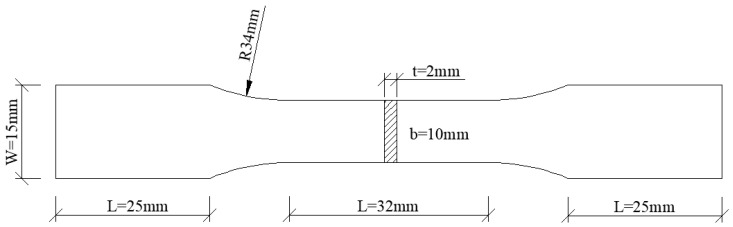
Dimensions for the tensile sample machined from the extruded sheet.

The tensile hardening behavior was characterized by performing a quasi-static uni-axial tensile test. Standard tensile specimens machined from the extruded plates in Figure 2 along extruding direction (ED) and transverse direction (TD) are shown in Figure 3. The test was conducted at ambient temperature with a strain rate of 6.7 × 10^−4^ s^−1^ and performed three times to ensure repeatability.

**Figure 4:**
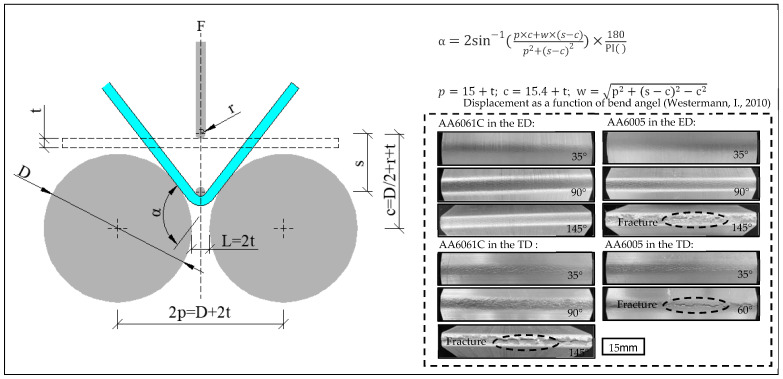
Schematic representation of the three-point bend and macro-surface of specimens bent to different angles in the ED and TD, respectively [26].

Bend test was carried out in a 10-ton universal testing machine according to VDA 238-100 [28]. A schematic of the bend test is shown in Figure 4, where r is the radius of the mandrel (i.e., 0.4 mm), D is the diameter of the supports (i.e., 30 mm) and t is the thickness of the extruded plates (2 mm). The square specimens, 60 mm both in length and width, were cut along the ED and TD directions in Figure 2. Before testing, the contact surface between the specimen and the roller or mandrel was lubricated to minimize the effect of friction. Specimens were bent to a displacement of 18 mm at a constant vertical speed of 20 mm/min. Force was logged as a function of vertical displacement. The bend strength and surface topography were evaluated to characterize the bending behavior. Specimens were also bent to different angles to evaluate the surface roughing and fracture behavior according to the previous force-displacement curves and formulas shown in Figure 4, which reveal the relationship between the bend angle and the displacement. The bend tests in the ED (i.e., bend axis parallel to TD) and TD (i.e., bend axis parallel to ED) were both performed to investigate the anisotropic bending behavior. 

### 2.3. Microstructure Characterization 

The microstructures of as-extruded plates and as-bent specimens were analyzed utilizing a series of characterization techniques. These methods include the electron backscatter diffraction (EBSD) to reveal the initial through-thickness microstructure (e.g., grain size, grain boundary, crystallographic texture) and its evolution during bend, optical microscopy (OM) to investigate the bend fracture through the thickness, and electric scanning microscopy (SEM) to characterize the particles distribution and surface topography of as-bent specimens. The OM specimens were polished using the standard metallography program and etched thereafter using a sodium hydroxide solution (NaOH). The samples for EBSD were ground, then they were mechanically polishing with sand papers and further electropolished in a solution of 10 ml perchloric acid and 90 ml ethanol at a voltage of 17 V for 15 s. All SEM imaging and EBSD measurements were performed in a Thermo Scientific Apreo 2 field emission scanning electron microscope. A step size of 3 µm was used for EBSD measurements, and the scan data were post-processed by the HKL-Channel 5 software. The scan data were cleaned using a single iteration of neighbour CI correlation to remove bad data points.

## 3. Results

### 3.1. Initial Microstructure and Tensile Behavior

**Figure 5:**
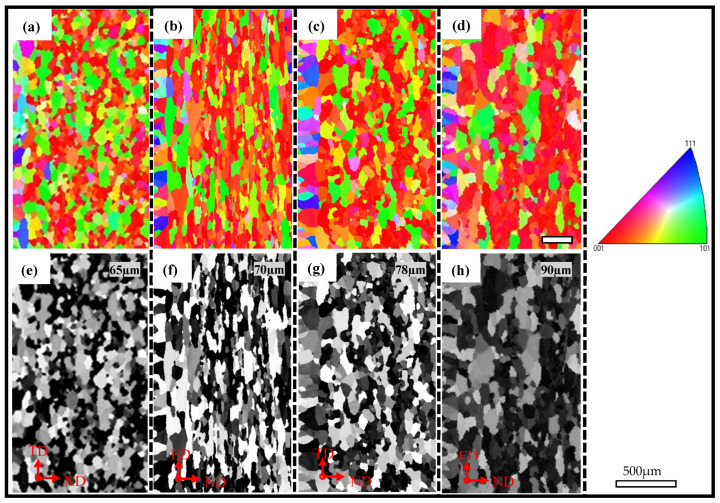
IPF and gray maps showing the through thickness microstructure: (**a**,**e**,**b**,**f**) AA6005; (**c**,**d**,**g**,**h**) AA6061C; (**a**,**e**,**c**,**g**) TD-ND Plane; (**b**,**f**,**d**,**h**) ED-ND plane.

**Figure 6:**
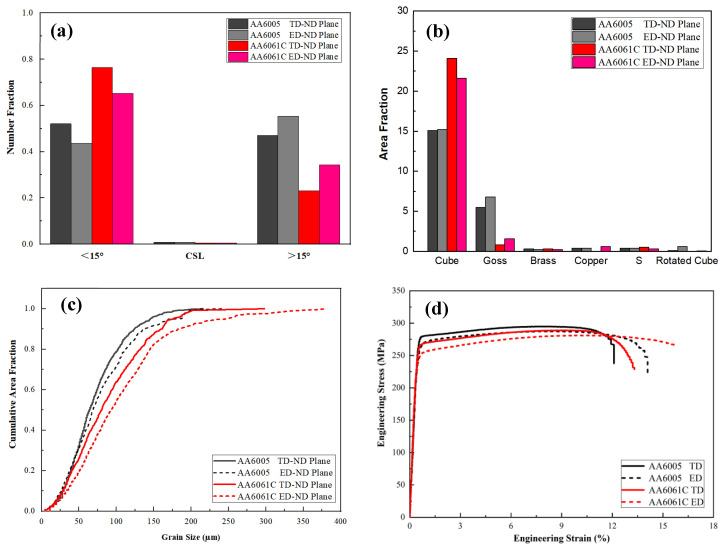
Comparison of grain boundary character distribution (**a**), area fraction of FCC texture components (**b**), grain size distribution (**c**), stress–strain behavior in tension test (**d**) of AA6005 and AA6061C.

The through-thickness texture measurements of the AA6005 and AA6061C materials along the two orthogonal TD-ND and ED-ND planes are shown in Figure 5. The grains tended to be elongated along the ED (see Figure 5f,h). Figure 6c shows that the grain size is relatively fine in AA6005. The average grain size along the TD-ND plane and the ED-ND plane is 65 µm and 70 µm for AA6005, and 78 µm and 90 µm for AA6061C, respectively. Figure 6a shows the grain boundary character distribution computed from the EBSD data, which are nearly independent on observed planes but closely related to the Mg/Si ratio. The number fraction of low-angle grain boundary (<15°) in AA6061C is 28% higher than that in AA6005 (i.e., 48%). Figure 6b reveals that the through-wall thickness texture is predominantly a cube texture, followed by a comparatively weak Goss texture for both alloys. Whereas AA6005 has less Cubic components (AA6005: 15%; AA6061C: 22%) and more Goss components (AA6005: 6.5%; AA6061C: 1.5%). Other texture components, such as Brass, Copper, S and Rotated Cubic are present at an extremely low fraction (less than 0.5%).

The typical tensile stress–strain curves are shown in Figure 6d. AA6061C has a comparatively low yield and high elongation. The 0.2% yield stress (or UTS) in the ED is 270 MPa (295 MPa) for AA6005 and 260 MPa (289 MPa) for AA6061C. The yield stress of both alloys (or UTS) is about 3% lower in the TD as compared to that in the ED (see Table 2). It is consistent with the values of the Schmid factor (i.e., 0.43 in the ED and 0.45 in the TD), which was computed from the EBSD data and was influenced by the texture components [29].

### 3.2. Intermetallic Particles Evolution

**Figure 7:**
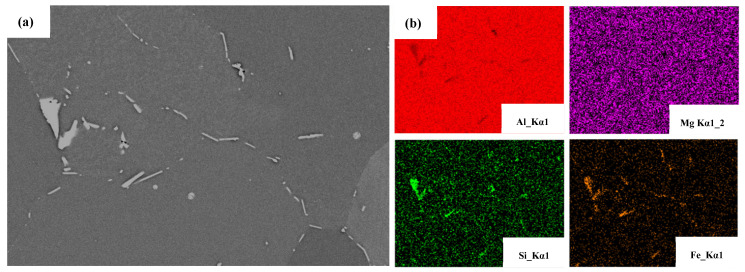
Backscattered electron diffraction (BSD) pattern (**a**) and elements mapping (**b**) of as-cast alloy AA6005.

**Figure 8:**
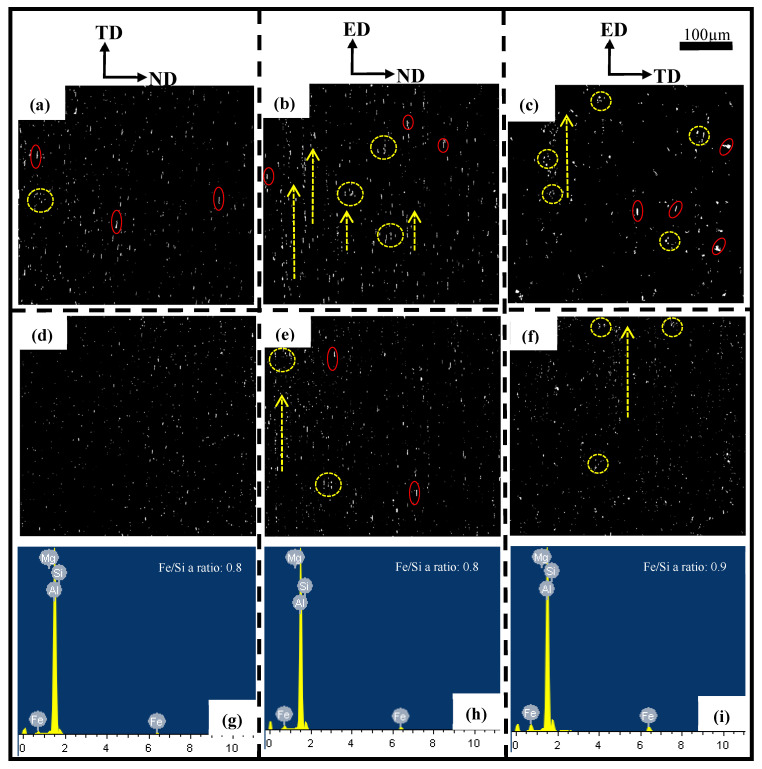
Backscattered SEM image and EDS for β-AlFeSi constituent particles: (**a**–**c**) AA6005 alloy; (**d**–**i**) AA6061C alloy; (**a**,**d**,**g**) TD-ND plane, (**b**,**e**,**h**) ED-ND plane, (**c**,**f**,**i**) ED-TD plane.

**Figure 9:**
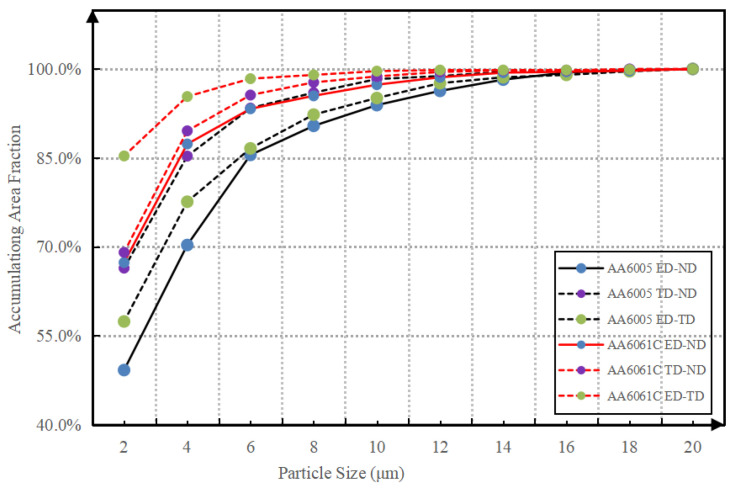
The accumulating area fraction of intermetallic particles relative to size in AA6005 and AA6061C.

The AlFeSi phase is formed in the interdendritic region during the final stage of solidification at the industrial cooling rate in AA6005 and AA6061 alloys [30,31]. Figure 7 shows the typical morphology of the AlFeSi phase and EDS elemental mapping. The AlFeSi phase in the as-cast state is identified by needle-like shapes or fragments of skeletons along the grain boundary. Figure 8 displays the distribution of the intermetallic particles on the TD-ND, ED-ND and ED-TD planes after extrusion. It is obvious that the hot extrusion changed the morphology and spatial arrangement of the AlFeSi phase, and its atom ratio of Fe/Si (0.8~0.9) is close to that of the β-Al5FeSi phase (see the EDS results in Figure 8). The brittle particles were broken to develop the mall clusters to within dozens of micrometers (as pointed by yellow circles) and string-type clusters to within hundreds of micrometers (as pointed by yellow arrows) along the ED on ED-ND and ED-TD planes, whereas the particles are randomly arranged on the TD-ND plane. The excessive Mg contributed to particles’ fragment during hot extrusion and resulted in the comparatively uniform distribution of particles in AA6061C (see Figure 8). In contrast, AA6005 with high Si content tended to develop dense particle clusters along the ED. Table 3 summarized the size, area fraction and number density of the particles in AA6005 and AA6061C. The two alloys both have comparatively high values of area fraction and average size of particles on the ED-ND plane. Figure 9 further shows the evolution of the particles’ accumulation of area fraction with size. The area fraction of particles smaller than 6 µm is 92% in AA6061C, 7% higher than that in AA6005. It is also apparent that the ED-ND plane shows more large particles in contrast to the TD-ND plane.

### 3.3. Bendability

**Figure 10:**
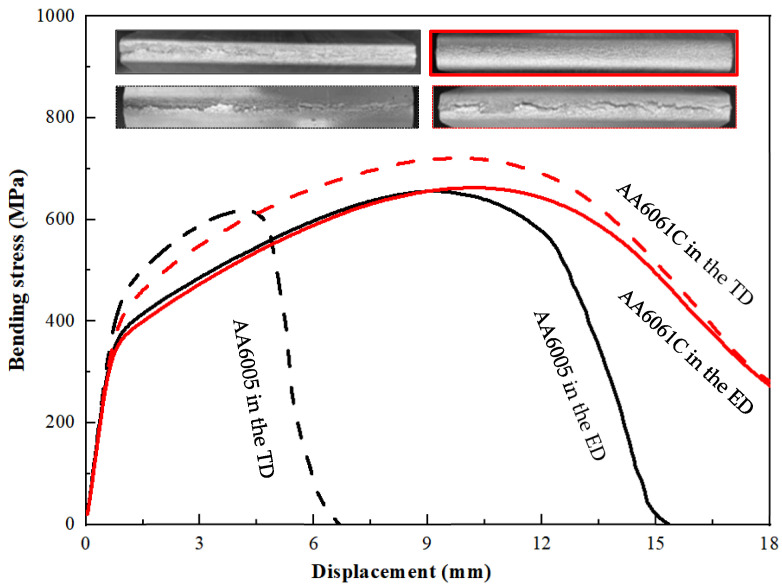
The stress–displacement curves obtained from the AA6005 and AA6061C alloys during bend in the ED and TD; the reproducibility of the results is good.

Figure 10 shows the stress-displacement curves measured when bending the AA6005 and AA6061C specimens in the ED and TD. The AA6061C specimens were deformed continuously in both directions, but the bend stress in the TD decreased prominently after bend strength because of the surface crack along the bend axis, whereas the AA6005 samples fractured rapidly after bend strength, especially in the TD. Furthermore, the curves in the TD show a higher work hardening rate before approaching the bend strength. The bend strength in the ED and TD is 618 MPa and 656 MPa for AA6005, and 663 MPa and 721 MPa for AA6061C. The bend angles corresponding to the bend strength in the ED and TD are 85° and 35° for AA6005, and 96° and 91° for AA6061C (see Table 2). It is obvious that AA6061C and AA6005 both show an anisotropic bend behavior, i.e., high values of work hardening rate and bend strength in the TD. AA6005 with excessive Si shows worse bendability in the TD and stronger anisotropy as compared to AA6061C with excessive Mg. 

### 3.4. Bending and Fracture Behavior in the ED

**Figure 11:**
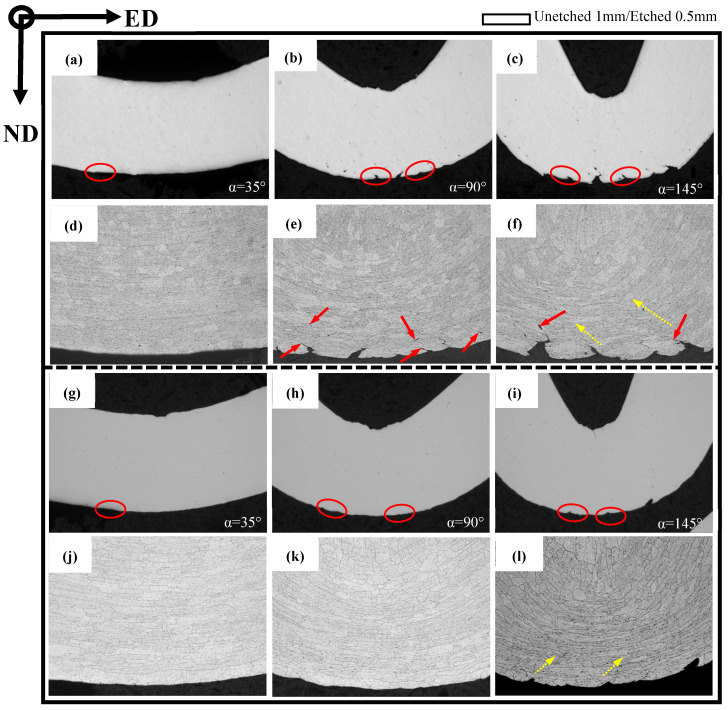
Through thickness optical micrographs of as-bent samples in the ED (unetched and etched): (**a**–**f**) AA6005 alloy; (**g**–**l**) AA6061C alloy; (**a**,**d**,**g**,**j**) 35°; (**b**,**e**,**h**,**k**) 90°; (**c**,**f**,**i**,**l**) 145°.

**Figure 12:**
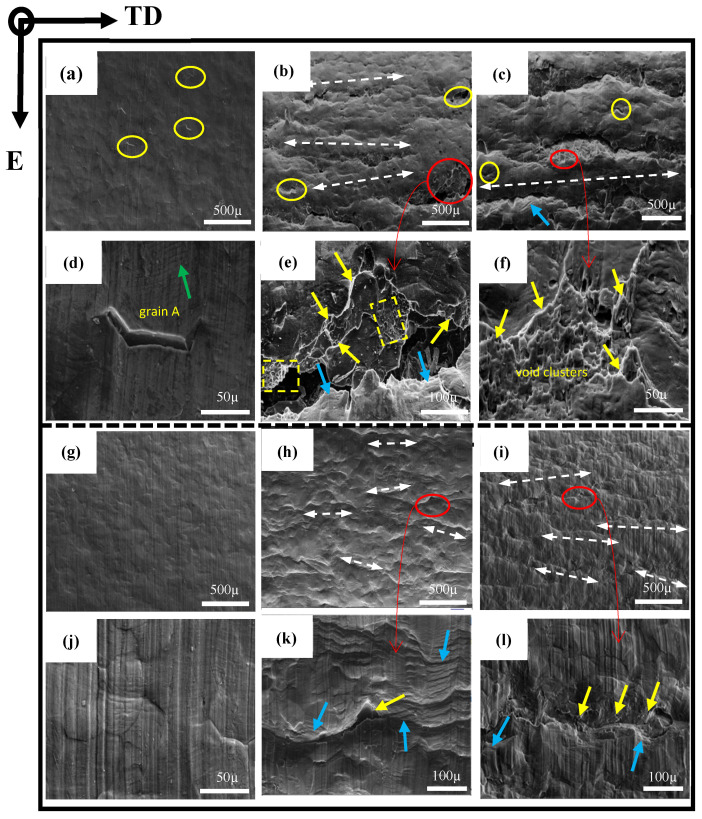
SEM micrographs and magnified views of the outer tensile surface of as-bent samples in the ED: (**a**–**f**) AA6005 alloy; (**g**–**l**) AA6061C alloy; (**a**,**d**,**g**,**j**) 35°, (**b**,**e**,**h**,**k**) 90°; (**c**,**f**,**i**,**l**) 145°.

In order to further investigate the bend behavior of AA6005 and AA6061C in the ED, the specimens were bent to different bend angles, i.e., 35°, 90° and 145°. Figure 11 and Figure 12 show the through thickness optical micrographs and SEM images of the bend surfaces, respectively. The slight surface undulations in Figure 11a,g and orange peel shown in Figure 12a,g are observed after bending to 35°. Figure 12d indicates that the relative sliding among grains caused the surface of “grain A” to move outwards and led to the grain boundary decohesion. As pointed out by the yellow circles in Figure 12a, grain boundary decohesion tended to occur in AA6005. The topography of the elongated surface was developed in a series of forms as increasing the bend angle, e.g., surface undulation (see red circles in Figure 11), slip steps (see blue arrows in Figure 12) and band-type ridges (see white arrows in Figure 12). The surface roughness and micro-cracks are comparatively easy to arise in AA6005. For instance, obvious undulations and micro-cracks were present after a 90° bend within a depth of several hundred micrometers (see red arrows in Figure 11). Surface cracks initiated within the valleys were propagated by several millimeters in length along the bend axis after a 145° bend (see Figure 12c). A couple of shear bands have propagated to inside along approx. 45° shear direction in both AA6005 and AA6061C after 145° bend (see yellow arrows in Figure 11), which promoted the surface roughing to develop the band-type ridges in Figure 12c,l. The fine dimples presented on site (see yellow arrows in Figure 12) indicate that the heterogeneously nucleated particles assisted the local crack process in both alloys. It is to be noted that local micro-cracks in AA6061C were never propagated to inside (see yellow arrows in Figure 11l and Figure 12l). 

### 3.5. Bending and Fracture Behavior in the TD

**Figure 13:**
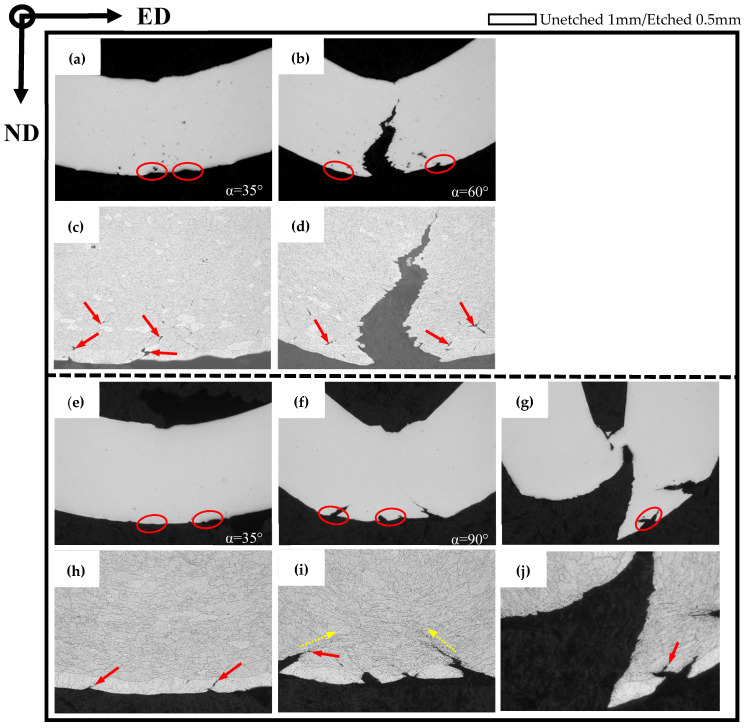
Through thickness optical micrographs of as-bent samples in the TD (unetched and etched): (**a**–**d**) AA6005 alloy; (**e**–**j**) AA6061C alloy; (**a**,**c**,**e**,**h**) 35°, (**b**,**d**,**f**,**i**) 90°; (**g**,**j**) 145°.

**Figure 14:**
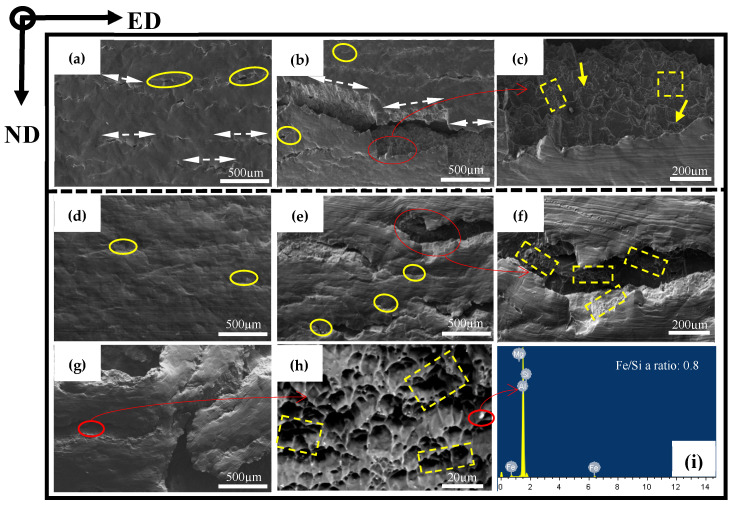
SEM micrographs and magnified views of the outer tensile surface of as-bent samples in the TD: (**a**–**c**) AA6005 alloy; (**d**–**i**) AA6061C alloy; (**a**,**d**) 35°; (**b**,**c**) 60°; (**e**,**f**) 90°; (**g**,**h**) 145°; (**i**) EDS result.

In order to investigate the anisotropic fracture behavior of AA6005 and AA6061C, Figure 13 and Figure 14 show the through-thickness optical micrographs and SEM images of the outer tensile surfaces in the TD. Depending on the bendability discussed in the previous section, the AA6005 samples were bent to the final bend angles of 35° and 60°, and the AA6061C samples were bent to the final bend angles of 35°, 90° and 145°. In contrast to the damage behavior in the ED, surface micro-cracks were easily stimulated and rapidly propagated in the TD. AA6005 shows numerous clusters of grain boundary relief on the elongated surface (see red arrows in Figure 14a) and some grain boundary decohesions underneath the surface (see red arrows in Figure 11c) after bending to 35° in the TD. These decohesions promoted strain localization and surface undulation (see red circles in Figure 13a). A macro-crack rapidly propagated along the bend axis and through the thickness after a 60° bend (see Figure 13b and Figure 14b). It is to be noted that the local shear performances assisted the connection between the neighboring minor cracks (see white arrows in Figure 14b). The crack path through the wall thickness in AA6005 tended to follow a zig-zag pattern (see Figure 13d). The corresponding fracture surfaces shown in Figure 14b,c reveal the polycrystalline structure. The fracture model in AA6005 is the predominately intergranular fracture in the TD. Figure 14c is the magnified view of the crack surface encircled in Figure 14b. The string-like voids (see yellow arrows in Figure 14c) and void clusters (see yellow rectangles in Figure 14c) near the grain boundary indicate that the heterogeneously nucleated particles assisted the intergranular fracture process. 

Excessive Mg is able to blunt the surface crack and cause a delay in fracture for AA6061C during bend in the TD. The AA6061C specimen after a 35° bend showed few grain boundary reliefs on the outer tensile surface (see Figure 13e and Figure 14d). Meanwhile, no signs of decohesions along the grain boundary are noticeable. Several macro surface grooves (i.e., valleys) are present along the elongated surface and are encircled in red in Figure 13f after a 90° bend. The cracks appear to initiate within these grooves and extend a few hundred micrometers through the thickness (see Figure 13i) and several millimeters roughly along the bend axis. It is important to note that the development of shear bands (see yellow arrows in Figure 13i) assisted the intense strain localization and grain boundary decohesion along the region ahead of the crack tip. At an even larger bend angle of 145° (see Figure 13g,j), the macro-crack had almost propagated through the entire thickness. As compared to the zig-zag pattern observed in AA6005, the crack path appears to be a more planar-flat type. Fine dimples (as pointed by the yellow rectangles) and comparatively ambiguous polycrystalline structures are observed on the fracture surface of AA6061C shown in Figure 14f,h. The EDS result (see Figure 14i) further indicates that β-AlFeSi particles also assist the crack propagation in AA6061C. It is apparent that the fracture model in AA6061C is the predominantly transgranular fracture but the predominantly intergranular fracture in AA6005.

## 4. Discussion

**Figure 15:**
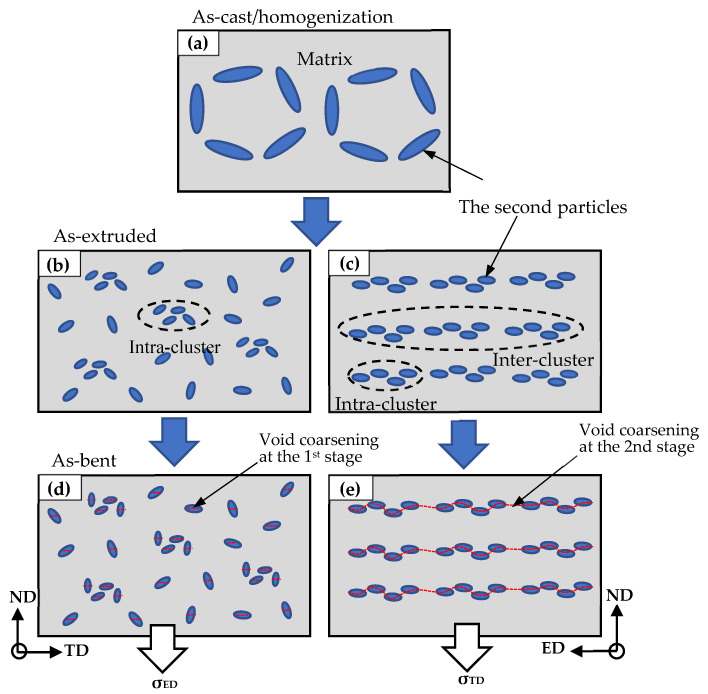
Schematic of the effect of anisotropic particle clusters in as-extruded materials on the bend damage behavior: (**a**) as-cast; (**b**) along the TD-ND plane; (**c**) ED-ND plane after extrusion; (**d**) along the TD-ND plane; (**e**) TD-ND plane after bend.

The primary particles (β-AlFeSi) after hot extrusion were broken and developed into anisotropic intra-clusters (see yellow circles in Figure 8) and inter-clusters along the extrusion direction (see yellow arrows in Figure 8). Figure 15 shows a schematic arrangement of these particle clusters. The inter-clusters assist the process of voiding and fracturing during bending when the loading is applied along the extrusion direction, which is discussed next. High-Si AA6005 exhibits more dense inter-clusters, while high-Mg AA6061C has a relatively small size and random distribution of particles. It is suggested that Mg with a comparatively low diffusion coefficient leads to a high value of flow stress during hot extrusion, which assists in particle cracking [32,33]. The two alloys both have a typical recrystallization texture, e.g., cubic and Goss. However, AA6005 has more Goss components and less cubic components than AA6061C. AA6061C has a comparatively large grain size and a high number fraction of low-angle grain boundary, which is closely linked with the dynamic recrystallization during hot extrusion [32,33,34].

AA6005 and AA6061C exhibit similar yield strengths and weak anisotropy during tension. Hannard et al. [35] also revealed that the onset of yielding and strain hardening behavior during tension do not significantly depend on the loading direction for the extruded 6005A. However, the bending anisotropy is obvious and depends on the Mg/Si ratio. Compared to the ED, the hardening rate is higher and the bendability is lower in the TD. The bend anisotropy observed in AA6005 is stronger than in AA6061C.

### 4.1. Bend Behavior in the ED

Recent studies have shown that bendability is limited by surface crack, which is usually preceded by particle cracking, voiding and the development of surface undulations along the outer tensile surface [15,18,36], which tends to occur when bending AA6005 specimens in the ED. A study on the bendability of AA6061 indicated that the ductile clad layer can accommodate large strains and enhances the bendability [13,25], which tends to occur when bending AA6061C specimens in the ED. AA6005 and AA6061C are designed to obtain the comparative microstructures (i.e., texture, particles distribution and grain boundary characteristics); thus, the specimens were further analyzed to investigate the effect of the microstructure on surface undulation and cracking.

#### 4.1.1. Strain Localization, Surface Undulation and Shear Banding

**Figure 16:**
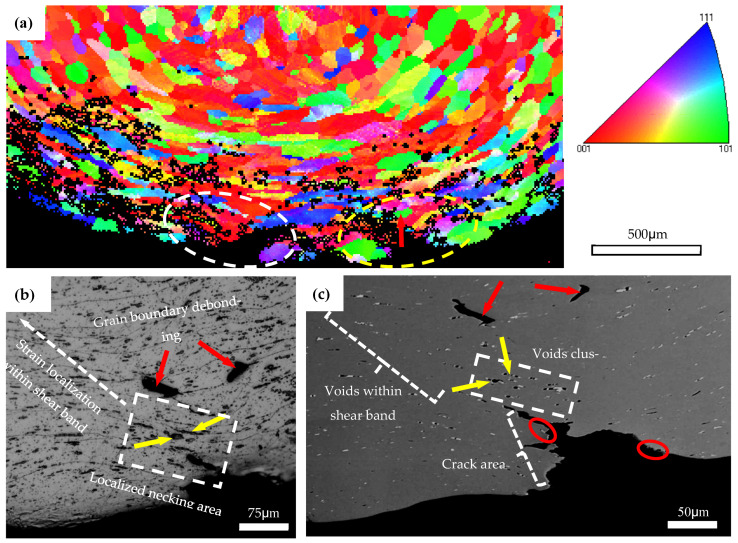
IPF map of AA6005 specimen bent to 145° in the ED (**a**), OM photo (**b**) and SEM map (**c**) of magnified view of region encircled in yellow in micrograph (**a**).

**Figure 17:**
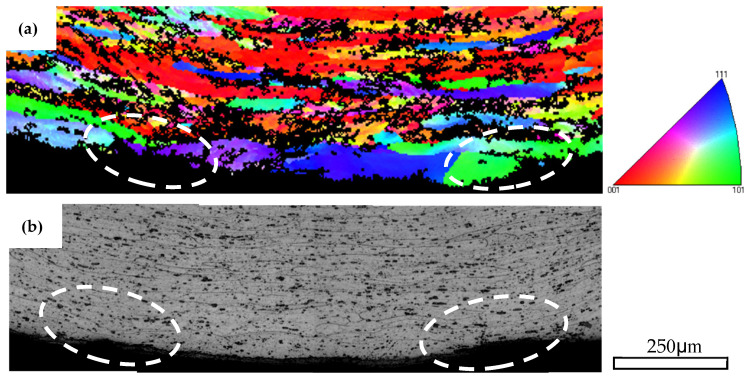
IPF map of AA6061C specimen bent to 145° in the ED (**a**) and the corresponding OM photo (**b**).

Figure 16 shows the IPF map near the outer tensile surface of AA6005 after a 145° bend in the ED and the corresponding OM and SEM images of the local area encircled in Figure 16a. Figure 17 shows the IPF and corresponding OM maps of AA6061 after a 145° bend. It is apparent that several through-thickness layers of grains show intense strain localization (see white circles in Figure 16a and Figure 17a). However, the region corresponding to the surface peaks is comparatively free of intense strain. Muhammad and co-workers [8] investigated the surface roughing behavior of an extruded AA6063 alloy during bend and revealed a similar phenomenon. It can be concluded that the through-thickness strain localization in the vicinity of the outer tensile region acts as a precursor to the formation of surface undulations (i.e., hills and valleys). The microstructure of AA6061C (i.e., low Goss component, random distribution of particles) promotes the strain uniform distribution along the outer tensile surface and causes a delay in surface roughing. A set of shear bands emanating from the surface’s low cusp regions are observed in AA6061C and can be seen to have propagated inside at a length of a couple of hundred micrometers (see yellow arrows in Figure 11i). It is suggested that surface undulation acts as a precursor to shear banding and is also promoted by the shear band as further increasing the bend angle. It is to be noted that the surface crack in AA6005 is also closely related to the shear band developing and surface roughing, which is discussed next.

#### 4.1.2. Effect of Particles on the Crack Initiation and Propagation

The previous results in Figure 11e,f indicate that heterogeneous nucleation grain boundary particles assist the cracking process in AA6005. Some signs of voids opened out and broken particles are noticeable along the crack path (see red circles in Figure 16c), which further indicates that the particles promoted the surface crack in AA6005. The cracks initiated within the valleys further propagated a localized necking area, which facilitated the development of shear bands along an approx. 45° shear direction (see yellow arrows in Figure 11e). The occurrence of crack propagation is impossible because void coarsening is limited during bending in the ED.

### 4.2. Fracture Behavior in the TD

The fracture of extruded Al-Mg-Si alloys in the TD is still not well understood, although recent publications show that it is limited by elongated intermetallic particles along the extrusion direction. Based on the results presented in previous sections, the bend fracture models in the TD for AA6005 and AA6061C both involve grain boundary ductility fracturing (GBDF) and transgranular fracturing, which are discussed next.

#### 4.2.1. Coarse Slip Distribution, Strain Localization and Decohesion along Grain Boundaries

**Figure 18:**
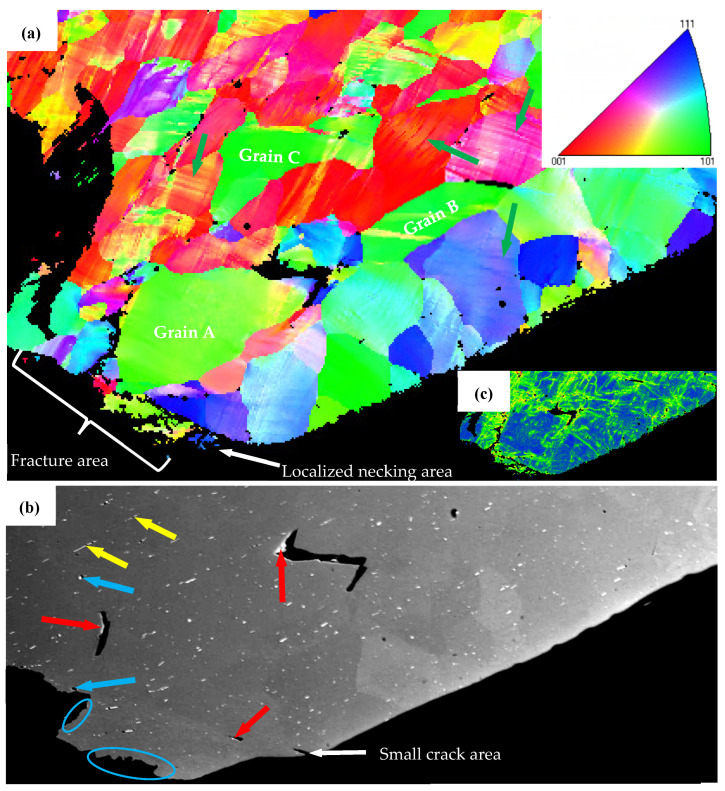
IPF map of AA6005 specimen bent to 60° in the TD (**a**), corresponding GND density (**b**) and SEM maps (**c**) of the local region in IPF map (**a**).

Figure 18 shows the local microstructure of the AA6005 specimen after a 60° bend in the TD by EBSD and SEM measurements. The coarse slip bands are apparent as banded regions of different colors within the parent grains (see green arrows in Figure 18a). It is interesting to note that the specific orientation and occurrence frequency of these slip bands are connected with the local crystallographic texture of the grain. For instance, the grain with a cube or near-cube orientation shows obvious slip bands at approx. 45° from the direction of the principal stress. The grain with near {111}(uvw) orientation shows less slip bands approx. 60° from the direction of principal stress, whereas the grain with the Goss orientation do not show the presence of slip bands. In other words, grain with a cube or near cube orientation, as “soft grain”, deforms easily to accommodate comparatively large strains. However, this is difficult to occur for a “hard grain” with a Goss orientation. The clustering of the soft and hard grains stimulates a strong heterogeneity of the strain distribution and further leads to decohesion along the grain boundary. It is apparent that typical grain clusters are observed in Figure 18a, i.e., hard grains (A, B and C) surrounded by its neighbors with cubic or {111}(uvw) orientation. As apparent from the geometrically necessary dislocation (GND) density map in Figure 18c, the localized strain within the soft grain encourages dislocations to pileup against the hard grain boundaries. It is interesting to note that grain boundary decohesion tends to occur at approx. 45° from the principal stress direction (see Figure 11, Figure 13, Figure 16 and Figure 18), which supports the maximum shear stress. Some signs of particles near the debonded grain boundaries are noticeable (see red arrows in Figure 16c and Figure 18b). It is suggested that decohesion along the grain boundary is the outcome of several competing micro-mechanisms, i.e., grain clusters, the grain boundary spatial arrangement and the heterogeneously nucleated particles.

#### 4.2.2. Influence of Particles Distribution on Fracture

β-AlFeSi is easily broken by tension deformation, owing to its diffuse interface with the aluminum matrix. It depends on the feature of particles (i.e., shape and aspect ratio) and loading direction during bending. For instance, needle-like particles with sharp edges and a high aspect ratio (see the yellow arrow in Figure 18b) can induce higher stress values as compared to globular particles. A few circle voids (see blue arrows in Figure 18b) and void clusters opened out (see blue circles in Figure 18b) are supposed to originate from the broken particles elongated along the ED. Figure 15 shows a schematic that reveals the effect of anisotropic particle clusters on the bend damage behavior. The primary particles are broken and elongated along the extrusion direction to develop the intra-cluster at a small scale (i.e., dozens of micrometers) and the inter-cluster at a large scale (i.e., hundreds of micrometers). Hence, the spatial distribution of particles after extrusion is featured by dense inter-clusters along the ED-ND plane (see Figure 15c) and a comparatively random arrangement along the TD-ND plane (see Figure 15b). When loading is applied along the ED, the stress field merging between particles leads to a high stress concentration and promotes the process of particles breaking and voids coarsening (see Figure 15e). The dense inter-clusters presented along the grain boundary further lead to grain boundary decohesion and follow predominately intergranular fracturing when bending the AA6005 specimen in the TD, i.e., the polycrystal structure on the fracture surface and dimple clusters near the grain boundary. This fracture mode is defined as the grain boundary ductile fracture (GBDF) in early publications [37,38]. The local shearing performances (see white arrows in Figure 14b) and grain boundary decohesion (i.e., some sharp grain boundaries with few dimples in Figure 14c) both assist the fracture propagation in nature when bending the AA6005 specimen in the TD.

**Figure 19:**
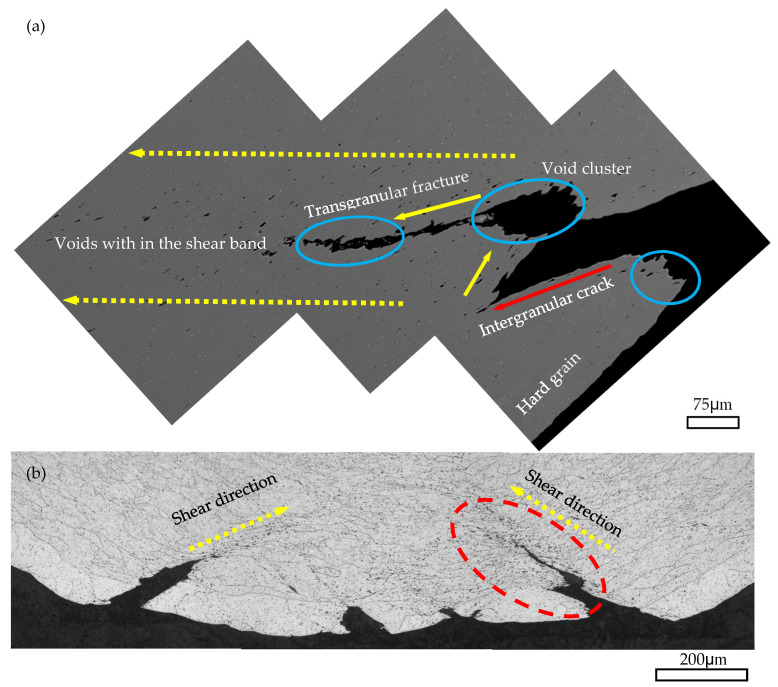
Through-thickness OM photos of AA6061C specimen bent to 90° in the TD (**b**) and SEM map (**a**) of the crack area encircled in red (**b**).

The model of GBDF is limited in AA6061C due to the small size and random distribution of the particles (see Figure 8 and Figure 9). Figure 19 shows the crack propagation inside the AA6061C specimen after bending to 90° in the TD. Based on the results shown in Figure 13 and Figure 14, the fracture of AA6061C in the TD is preceded by particle cracking, voiding and the development of a shear band. The cracking behavior of AA6061C in the TD is similar to that of AA6005 in the ED, i.e., cracks within the valleys, grain boundary decohesion and void clusters opened out along the crack path (see blue circles in Figure 19a). When the bending axis is parallel to the ED, broken particles and void nucleation are easier to occur within the shear band (see Figure 19a). The void’s coarsening and clustering shortens the space between neighbors as the shear band propagates ahead and stimulates the transgranular fracture. In contrast, the crack propagation is limited during the bending of AA6005 in the ED because the void’s coarsening and clustering are blunted when the loading is applied perpendicular to the inter-clusters (see Figure 15d).

### 4.3. The Overall Sequence of Microstructural Events to Damage Behavior

**Figure 20:**
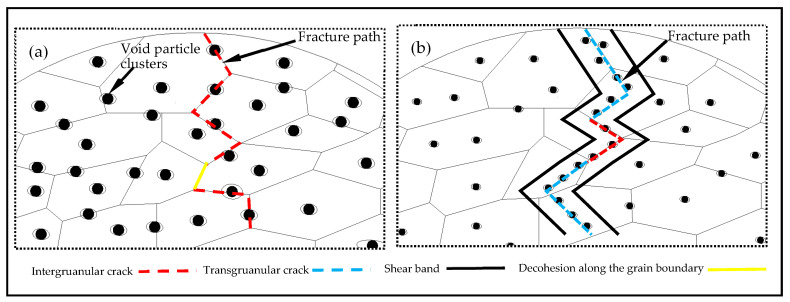
Schematic of fracture model during bend in the TD: (**a**) AA6605; (**b**) AA6061C.

Based on the discussion above, the damage behavior in bend is decided by the loading direction, which affects the stress intensity field during stressing and the Mg/Si ratio, which in turn affects the microstructure, e.g., texture, particles, grain boundary. During bending in the ED, no signs of crack propagation are noticeable in AA6005 and AA6061C. The high-Mg AA6061C shows near unlimited bendability. However, surface cracks were present in AA6005 and were preceded by particle cracking, voiding, shear banding and the development of surface undulation. The two alloys both show worse bendability but comparatively different fracture behaviors in the TD. Figure 20 shows the schematic of the fracture model in AA6005 and AA6061C during bending in the TD. The fracture model in AA6005 (see Figure 20a) is a predominately intergranular fracture in nature, with some areas exhibiting grain boundary decohesion, which is caused by local micro-texture (i.e., Goss, cubic or near cubic). However, the fracture model in AA6061C (see Figure 20b) is a predominately transgranular fracture propagated by a shear band in nature with some areas exhibiting intergranular cracking caused by heterogeneously nucleated particles.

## 5. Conclusions

In the present work, the two alloys with different Mg/Si ratios were extruded to the 2 mm thick sheets to study their bendability and anisotropic fracture behavior. AA6005 is the high-Si alloy and AA6061C is the high-Mg alloy. The analysis techniques, e.g., tensile test, bend test, EBSD texture measurement, optical and scanning electron microscopy were used to examine the bendability and fracture behavior of the two alloys. The main purpose was to reveal the relationship between the microstructures (i.e., micro-texture, particles, grain boundary orientation), bend behavior (i.e., surface topography development, crack initiation, shear banding) and fracture behavior (i.e., macro-crack propagation, grain boundary decohesion). Some important results are summarized below.

1. AA6005 and AA6061C alloys both have a typical recrystallization texture, which is characterized by the cube and Goss texture component. However, excessive Mg is beneficial for obtaining more cubic components, low-angle grain boundaries and randomly arranged particles in AA6061C. In contrast, excessive Si leads to more Goss components, large-angle grain boundaries and dense particle clusters in AA6005.
2. The grain with a Goss or {111}(uvw) orientation, as a “hard grain”, is difficult to deform. In contrast, the grain with a cubic or near cubic orientation, as a “soft grain”, is easy to deform to accommodate a large strain. The soft and hard grains clustering stimulated strong strain heterogeneity, and promoted surface ridging and decohesion along the grain boundary.
3. β-AlFeSi in as-cast alloys is broken after hot extrusion to develop the intra-clusters within dozens of micrometers and inter-clusters within hundreds of micrometers along the extrusion direction, which lead to anisotropic bend behavior. AA6005 has more dense particle clusters and shows stronger bend anisotropy and worse bendability in contrast to AA6061C.
4. When bending in the ED, the development of the surface topography in AA6061C is preceded by orange peel, surface undulation, and banded-type ridges. The surface ridging behavior is promoted by the strain localization of several layers of grains in the vicinity of the elongated surface. The microstructure characteristics in AA6005, i.e., more Goss component, large angel grain boundaries and dense particle clusters, promote surface roughing and cracks in AA6005. The micro-cracks are initiated within valleys and further promote strain localization and surface roughing. However, the occurrence of crack propagation is impossible because void coarsening is limited during bending in the ED.
5. When bending in the TD, the fracture mode in AA6005 is predominately intergranular fracture, which is affected by heterogeneous nucleation grain boundary particles. However, AA6061C showed a predominately transgranular fracture, which was effected by shear band development and void coarsening.

## Figures and Tables

**Table 1:** Chemical compositions of AA6005 and AA6061C alloys.

Alloy	Component (mass%)								Mg/Si at. Ratio	Mg + Si (mass%)	Excessive Element
	Si	Fe	Cu	Mn	Mg	Cr	Ti	Al			
AA6005	0.80	0.18	×	×	0.55	×	0.020	Bal.	0.7	1.35	Si-rich
AA6061C	0.55	0.18	×	×	0.80	×	0.020	Bal.	1.5	1.35	Mg-rich

**Table 2:** The tensile and bending properties for extruded AA6605 and AA6061C alloys.

Alloy	Deformation Direction	Yield Strength (MPa)	Tensile Strength (MPa)	Total Elongation (%)	Normalized Bend Angle (°)	Bend Strength (MPa)
AA6005	in the ED	270	295	12.0%	85	618
	in the TD	262	285	13.3%	35	656
AA6061C	in the ED	260	289	12.8%	96	663
	in the TD	252	280	15.6%	91	721

**Table 3:** The quantitative statistics data of the second phase particles in the AA6005 and AA6061C alloys.

Alloy	Observed Planes	Area Fraction [%]	Average Length [µm]	Max. Length [µm]	Number Density [mm^2^]
AA6005	ED-TD	0.8	2.1	16	3.05 × 10^3^
	TD-ND	0.9	2.7	18	2.87 × 10^3^
	ED-ND	0.8	2.6	15	2.71 × 10^3^
AA6061C	ED-TD	0.7	1.5	10	3.21 × 10^3^
	TD-ND	0.8	2.1	15	3.39 × 10^3^
	ED-ND	0.7	1.0	12	3.80 × 10^3^

## Data Availability

Not applicable.
